# Evaluating chain-of-thought prompting in a GPT chatbot for BCID2 interpretation and stewardship: how does AI compare to human experts?

**DOI:** 10.1017/ash.2025.10059

**Published:** 2025-07-11

**Authors:** Daniel M. Tassone, Matthew M. Hitchcock, Connor J. Rossier, Douglas Fletcher, Julia Ye, Ian Langford, Julie Boatman, J. Daniel Markley

**Affiliations:** 1 Division of Infectious Diseases, Department of Medicine, Central Virginia VA Health Care System, Richmond, VA, USA; 2 Virginia Commonwealth University, School of Pharmacy, Richmond, VA, USA; 3 Division of Infectious Diseases, Department of Medicine, Virginia Commonwealth University School of Medicine, Richmond, VA, USA; 4 Department of Health Informatics, Central Virginia VA Health Care System, Richmond, VA, USA

## Abstract

**Background:**

Rapid molecular diagnostics, such as the BIOFIRE® Blood Culture Identification 2 (BCID2) panel, have improved the time to pathogen identification in bloodstream infections. However, accurate interpretation and antimicrobial optimization require Infectious Disease (ID) expertise, which may not always be readily available. GPT-powered chatbots could support antimicrobial stewardship programs (ASPs) by assisting non-specialist providers in BCID2 result interpretation and treatment recommendations. This study evaluates the performance of a GPT-4 chatbot compared to ASP prospective audit and feedback interventions.

**Methods:**

This prospective observational study assessed 43 consecutive real-world cases of bacteremia at a 399-bed VA Medical Center from January to May 2024. The GPT-chatbot utilized “chain-of-thought” prompting and external knowledge integration to generate recommendations. Two independent ID physicians evaluated chatbot and ASP recommendations across four domains: BCID2 interpretation, source control, antibiotic therapy, and additional diagnostic workup. The primary endpoint was the combined rate of harmful or inadequate recommendations. Secondary endpoints assessed the rate of harmful or inadequate responses for each domain.

**Results:**

The chatbot had a significantly higher rate of harmful or inadequate recommendations (13%) compared to ASP (4%, *p* = 0.047). The most significant discrepancy was observed in the domain of antibiotic therapy, where harmful recommendations occurred in up to 10% (*p* <0.05) of chatbot evaluations. The chatbot performed well in BCID2 interpretation (100% accuracy) but provided more inadequate responses in source control consideration (10% vs. 2% for ASP, *p* = 0.022).

**Conclusions:**

GPT-powered chatbots show potential for supporting antimicrobial stewardship but should only complement, not replace, human expertise in infectious disease management.

## Manuscript background

The rapid identification of pathogens in bloodstream infections is essential for improving patient outcomes and antibiotic optimization.^1^ Culture-based techniques for organism identification and susceptibility testing are time-consuming, which may delay appropriate antimicrobial therapy in multi-drug-resistant infections. Recently, rapid molecular diagnostic tools, such as the BIOFIRE® Blood Culture Identification 2 (BCID2) panel (bioMérieux), have emerged as promising solutions, significantly reducing the time to pathogen identification.^2^ BCID2 is a multiplex PCR assay that identifies 43 targets, including 26 bacteria, 7 yeast, and 10 antimicrobial resistant genes, with results typically available in an hour after a positive Gram stain is reported.^3^ However, accurate interpretation of these results to optimize antibiotic therapy often requires specialized expertise from Infectious Disease (ID) physicians or antimicrobial stewardship programs (ASPs), as evidence suggests that rapid diagnostic tests are associated with decreased mortality and length of stay, but only in combination with ASP interventions.^4,5^

ASPs are critical in optimizing antibiotic use across healthcare settings, but these interventions are resource-intensive and time-sensitive.^6,7^ Currently, there is a demand for ID specialty care that exceeds the supply and is expected to worsen.^8^ Compounding this issue, microbiologic and other diagnostic results may be reported at inconsistent and non-standardized times when immediate ASP coverage is unavailable. These constraints underscore the need for validated tools to support non-specialist healthcare providers in stewardship efforts, particularly in facilities with limited ASP resources.

Artificial Intelligence (AI) offers a potential solution to bridge these gaps. Leveraging AI to assist ASPs, particularly in prospective audit and feedback (PAF) interventions, could enable faster, data-driven support for frontline providers.^9^ A branch of AI known as Large Language Models (LLMs) holds significant potential for applications in healthcare.^10^ One widely recognized application of LLMs is Generative Pretrained Transformer (GPT)-powered chatbots.^11^ ChatGPT by OpenAI, first released in 2022, was the first of many publicly available LLM-powered chatbots.^12^ While not specifically designed for medical use, potential areas for applications include provider documentation of patient encounters, automated patient responses, clinical decision-making, and diagnostic interpretation.^13–18^ GPT-powered chatbots enable users to converse using speech or written natural language, known as prompting, to receive responses to complex queries.^19^ However, validating the chatbot’s response to a user’s prompt for healthcare use is crucial, as they have an inherent tendency to “hallucinate,” generating responses that appear credible but may be incorrect. LLMs that power chatbots generate responses by predicting the next words based on patterns in their training data, rather than verifying factual accuracy. This can lead to hallucinations, which are misleading or off-topic responses that could be harmful in clinical decision-making if not recognized.^20^ Despite their potential, few studies have evaluated the utility of GPT-powered chatbots in supporting direct ID consultations or pharmacotherapy recommendations, underscoring the need for rigorous validation before integration into clinical practice.^18,21–23^

At our institution, ASP PAF interventions are routinely conducted following the publication of BCID2 results to ensure prompt and appropriate antimicrobial selection, but may not be able to respond to all results in real-time. This proof-of-concept study serves as an initial step in validating ChatGPT-4 for potential future use to assist non-ID providers in interpreting BCID2 results and providing initial treatment recommendations in a timely manner. We attempted to minimize hallucinations from the chatbot by implementing both “chain-of-thought” (COT) prompting, and external knowledge integration (EKI). COT prompting is a technique where the user guides the chatbot’s responses in a step-by-step thought process. EKI provides the chatbot with access to data beyond its pretrained model to improve its response. We uploaded reference documents developed with local and national guidelines for the treatment of bacteremia to enhance the chatbot’s recommendations.

The goal of this study was to assess the performance of the ASP PAF intervention against the chatbot in real-world clinical settings for patients with BCID2 results. This was achieved by evaluating the quality of BCID2 interpretations and treatment recommendations for the two groups. By undertaking this study, we hope to assess AI’s viability for future use as a supplementary tool for non-specialists to optimize antimicrobial therapy in patients with bacteremia.

## Methods

### Primary endpoint

The primary end point of this study was the combined rate of recommendations that could cause patient harm by the chatbot versus ASP PAF interventions for patients with BCID2 results. This included incorrect BCID2 result identification and harmful or inadequate antibiotic recommendations. Secondary endpoints included individual assessment of the rate of harmful or inadequate recommendations for BCID2 interpretation, source control considerations, antibiotic therapy, and additional diagnostic workup.

### Study design and participants

This prospective, observational study was conducted from January to May 2024 at a 399-bed tertiary care Veteran Affairs Medical Center in Richmond, Virginia. It included all adult in patients and out patients with positive BCID2 results during the study period.

**Figure 1.**
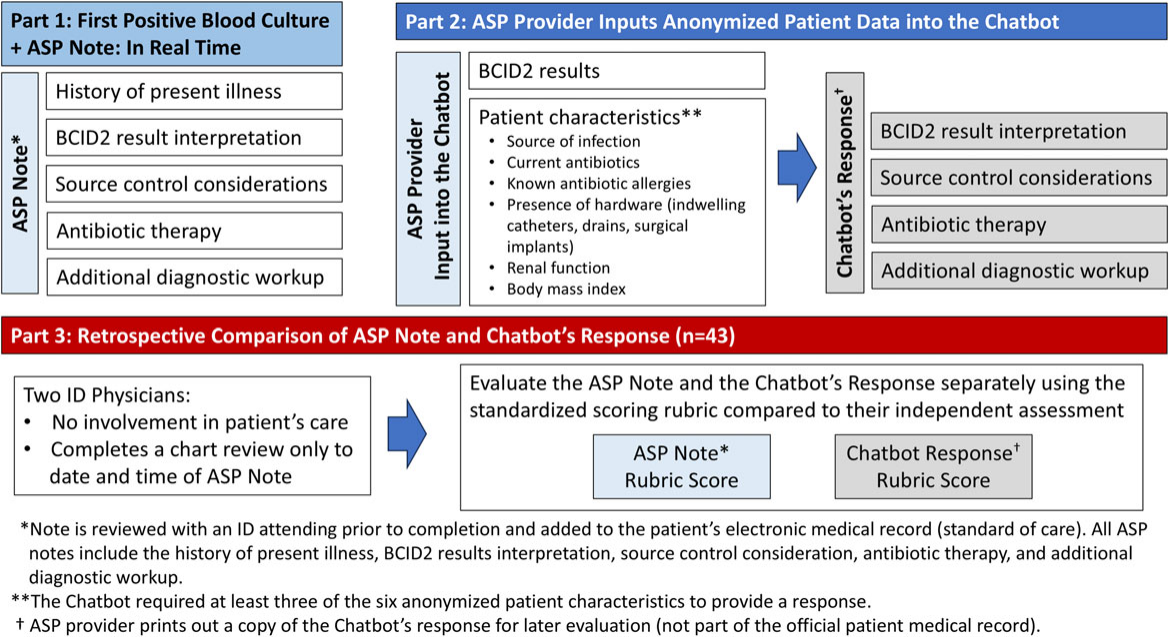
Study design. Illustrates the study design, outlining three key phases. Part 1 details the antimicrobial stewardship program (ASP)’s real-time documentation of BCID2-positive blood culture cases, including history of present illness, BCID2 interpretation, source control considerations, antibiotic therapy, and additional diagnostic workup. Part 2 describes the ASP provider inputting anonymized patient data into the chatbot, which then generates a response structured similarly to the ASP note. Part 3 involves a retrospective comparison of the ASP note and chatbot response by two independent ID physicians, using a standardized scoring rubric. The diagram visually represents the workflow from clinical documentation to AI evaluation and comparison.

The ASP team was comprised of two Board-Certified Infectious Diseases Clinical Pharmacists and two ID-trained physicians. At this institution, ASP PAF interventions follow a structured workflow initiated by an alert in the electronic health record (EHR) of a positive Gram stain from a blood culture. BCID2 results are generally reported an hour after the positive Gram stain. The ASP pharmacist then determines appropriate antimicrobial therapy and documents the PAF as blood culture notification note in the EHR (Figure 1 and to Supplementary Figure S1 for an example of mock ASP Blood Culture Notification Note).

### Chain-of-thought prompting and external knowledge integration

This study utilized a GPT-4 chatbot with COT prompting to generate patient-specific treatment recommendations based on BCID2 results and anonymized patient data. COT prompting was used to enhance the chatbot’s reasoning process, attempting to produce more structured and clinically relevant responses. Furthermore, we aimed to utilize EKI by uploading reference documents developed with local and national guidelines for the management of bacteremia.^24–31^ This allowed the chatbot to incorporate institutional protocols and best practices into its recommendations. By leveraging both COT prompting and EKI, the chatbot could generate responses aligned with the four key domains of the ASP note: BCID2 results interpretation, source control considerations, antibiotic therapy, and additional diagnostic workup (refer to Supplementary Table S1–S2 for our COT prompts and EKI reference documents).

Patient data entry followed HIPAA’s 18-point de-identification standard to ensure anonymity. The patient’s ASP note was not used as input into the chatbot. Instead, the chatbot required BCID2 results and at least three of six patient characteristics before generating recommendations (Figure 1). The chatbot’s output, structured as a conversation, was printed for retrospective analysis but excluded from clinical decision-making and is not part of the patient EHR (an example of a mock conversation is included in Supplementary Table S3).

### Evaluation

The evaluation team consisted of four ID physicians from our hospital who were not involved in the patient’s management. Each case was assigned separately to two evaluators for independent review. The evaluators were provided with a printed copy of the ASP note and instructed to retrospectively and independently review the patient’s EHR up to the date and time of the completion of the ASP’s Blood Culture Notification Note. They were also provided with a printed copy of the chatbot’s responses. The two evaluators then independently applied a rubric that evaluated how the chatbot’s and the ASP’s note compared to their own retrospective assessment, which represents this study’s “gold standard” (Figure 1). No cases were excluded due to discrepancies between evaluators to reduce bias and represent real-world provider management styles and treatment preferences.

**Figure 2.**
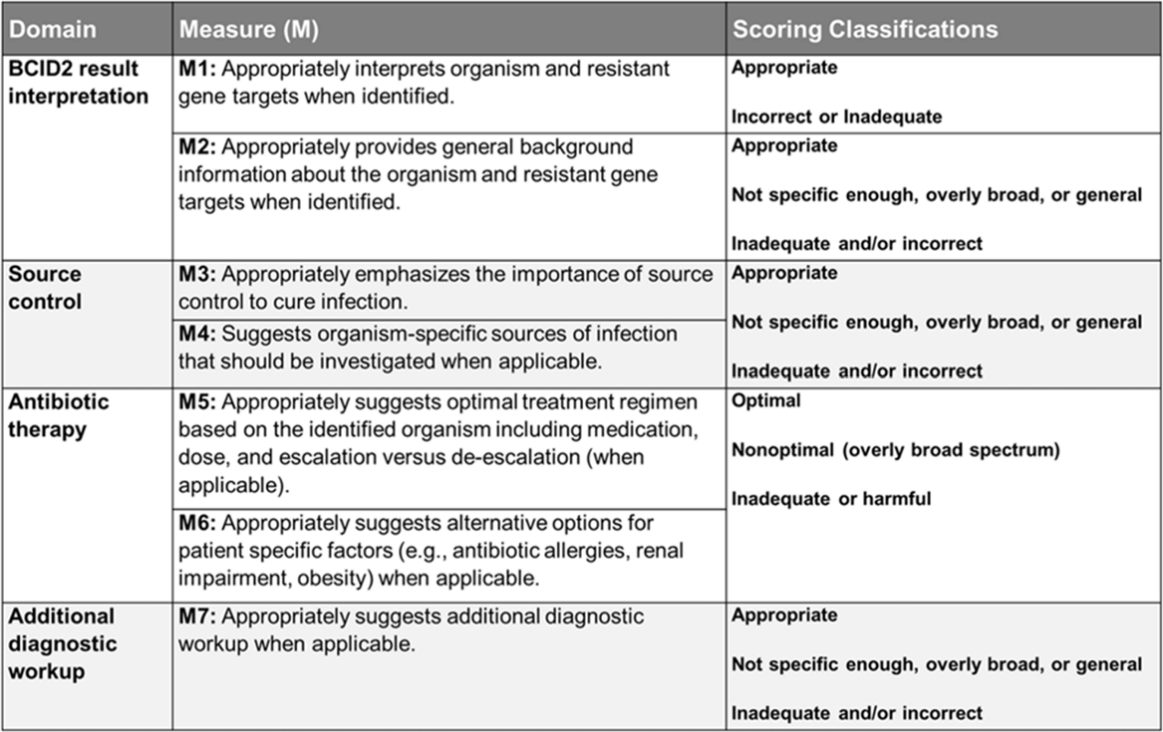
Evaluation rubric. Displays the evaluation rubric used to assess the accuracy and appropriateness of BCID2 result interpretation, source control considerations, antibiotic therapy recommendations, and additional diagnostic workup. The table outlines seven measures (M1–M7) across four domains, specifying scoring classifications such as appropriate, non-optimal, overly broad, or inadequate/harmful responses. This rubric provides a structured framework for evaluating the performance of ASP and chatbot recommendations.

The evaluation rubric contained four domains with a total of 7 individual measures (Figure 2). Measures were assessed into one of three categories: optimal, non-optimal or broad, and inadequate or harmful responses. Measures 1, 5, and 6 carry high clinical significance when assessing for potential patient harm, and any inadequate or harmful score in these measures was considered a failure overall (refer to Supplement Table S4 for a comprehensive table for domain and measure definitions).

### Statistical analyses

The statistical analysis was designed to compare the rate of recommendations that could cause patient harm by the chatbot versus ASP PAF interventions. The primary end point combined measures 1, 5, and 6 from the domains of BCID2 Results Interpretation and Antibiotic Recommendations due to these having the highest clinical significance when assessing for potential patient harm. If one or more of the above 3 measures were categorized as harmful by a single evaluator, then the entire case was considered harmful. The secondary endpoints individually assessed the rate of harmful or inadequate recommendations for each domain and individual measure for all 84 evaluations. Fisher’s exact test was used to assess differences in counts of harmful or inadequate responses between the two groups with a *P* ≤ .05. All analysis was completed using R software (v4.4.2).

### Ethics

This study was approved by our local Investigational Review Board. No written informed consent was required. All patient data entry followed HIPAA’s 18-point de-identification standard to ensure anonymity. No chatbot output was used to direct patient care or documented in the patient’s EHR.

## Results

A total of 43 cases of bacteremia were analyzed in this study and a total of 84 evaluations were included (2 cases only had one evaluator). Most patients were male (98%) with a median age of 74 years (Table 1). The primary end point of the combined rate of evaluations classified as harmful or inadequate was significantly higher in the chatbot group (13%) compared to the ASP PAF interventions (4%), with a *P* value of .047 (Table 2). In the ASP group, two evaluations in the domain of BCID2 Result Interpretation (M1: Organism Identification Accuracy) were classified as inadequate, whereas the chatbot had none. This discrepancy stemmed from an error in the ASP note template, where *Klebsiella aerogenes* was mistakenly selected instead of *Klebsiella pneumoniae*. However, the recommended treatment remained appropriate, and no actual patient harm occurred. For Antibiotic Therapy and Additional Diagnostic Workup (M5 and M6), the chatbot had eight cases with a total of 11 harmful or inadequate evaluations, compared to only one case in the ASP group (Table 3). The single harmful classification in the ASP group was in M6, where one evaluator rated the case as inadequate. This involved a patient with a coagulase-negative *Staphylococcus* sp. [CoNS] in 1/4 bottles presumed to be a contaminant. The evaluator classified the recommendation as inadequate because no alternative treatment was suggested in case the CoNS represented a true bloodstream infection.

**Table 1.** Characteristics of the study population (*n* = 43)

Patient characteristics (%)			Source (%)			Organism (%)			Resistant genes (%)	
Age (years)	74		Urogenital	16 (37)		Staphylococcus	19 (44)		Mec A/C + MREJ	1 (2)
Sex	41 (98)		Contaminate	8 (19)		Enterobacterales	16 (37)		Mec A/C	6 (14)
Antimicrobial	7 (16)		Pulmonary	6 (14)		Negative result	3 (7)		CTX-M	2 (5)
Allergy			Intra-abdominal	3 (7)		Streptococcus	3 (7)			
			Osteomyelitis	3 (7)		Enterococcus	2 (5)			
			Soft skin tissue infection	3 (7)		Nonfermenting	1 (2)			
			Line infection	1 (2)		Yeast	1 (2)			
			Postoperative	1 (2)						

Presents the characteristics of the study population, including patient demographics, sources of infection, identified organisms, and detected resistant genes. It provides an overview of the clinical and microbiological profile of the included cases, categorizing patient factors, infection origins, and relevant resistance markers.

**Table 2.** Comparison of ASP PAF interventions and chatbot performance

End point	Measure	Evaluation classification	ASP	Chatbot	*p* value*
**Primary safety outcome**	M1+M5+M6	Safe	96%	87%	
		Harmful or inadequate	4%	13%	.047
**Domain**	**Measure**	**Evaluation classification**	**ASP**	**Chatbot**	*p* value*
**BCID2 result interpretation**	M1	Adequate	98%	100%	
		Harmful or inadequate	2%	0%	.497
	M2	Optimal	96%	87%	
		Non-optimal	0%	12%	
		Inadequate	4%	1%	.001
**Source control considerations**	M3	Optimal	92%	88%	
		Non-optimal	8%	10%	
		Inadequate	0%	2%	.534
	M4	Optimal	92%	76%	
		Non-optimal	6%	14%	
		Inadequate	2%	10%	.022
**Antibiotic therapy**	M5	Optimal	94%	63%	
		Non-optimal	6%	29%	
		Inadequate	0%	8%	.001
	M6	Optimal	98%	77%	
		Non-optimal	1%	13%	
		Inadequate	1%	10%	.022
**Additional diagnostic workup**	M7	Optimal	81%	75%	
		Non-optimal	16%	20%	
		Inadequate	4%	5%	.680

*The *P* value represents the difference in the rate of harmful or inadequate responses between the ASP and the Chatbot. Abbreviations: **M1:** Appropriately interprets organism and resistant gene targets when identified; **M2:** Appropriately provides general background information about the organism and resistant gene targets when identified; **M3:** Appropriately emphasizes the importance of source control to cure infection; **M4:** Suggests organism-specific sources of infection that should be investigated when applicable; **M5:** Appropriately suggests optimal treatment regimen based on the identified organism including medication, dose, and escalation versus de-escalation (when applicable); **M6:** Appropriately suggests alternative options for patient-specific factors (e.g., antibiotic allergies, renal impairment, obesity) when applicable; **M7:** Appropriately suggests additional diagnostic workup when applicable. Compares the performance of ASP PAF interventions and a GPT-powered chatbot in evaluating BCID2 results and providing treatment recommendations. The table presents results for the primary end point, measuring the combined rate of harmful or inadequate recommendations (M1+M5+M6), as well as secondary endpoints across four domains: BCID2 result interpretation, source control considerations, antibiotic therapy, and additional diagnostic workup. Each domain includes specific measures with classification into optimal, non-optimal, or harmful/inadequate responses for both ASP and chatbot groups. Statistical significance (p-values) is provided for differences in harmful or inadequate responses between ASP and chatbot recommendations.

**Table 3.** Characterization of cases with non-optimal and harmful chatbot responses for antibiotic therapy recommendations

Category (*n*)	Reason*	Percent (*n*)	Comment
Non-optimal (15)	Overly broad	100% (15)	Due to likely blood culture contaminate or provider preference
Harmful (8)	Inadequate coverage	50% (5)	Polymicrobial infections or sources
	Incorrect dose	20% (2)	Incorrect renal impairment dosing
	Unclear recommendation	10% (1)	Unclear to discontinue current treatment before starting new treatment
	Misdiagnosis	10% (1)	Ignored co-infection

*Each case can have multiple reasons to cause non-optimal or harmful/inadequate classifications. Presents a classification of chatbot-generated antibiotic therapy recommendations, distinguishing between non-optimal and harmful responses. It categorizes errors based on reasoning, such as overly broad antibiotic coverage, inadequate coverage, incorrect dosing, unclear recommendations, and misdiagnosis. The table provides insight into the types of errors the chatbot made in antimicrobial selection, highlighting key areas where AI-assisted decision-making may require further refinement to align with clinical best practices.

The secondary analysis examined the rate of harmful or inadequate responses within each individual domain. In BCID2 Result Interpretation, M1 showed a 2% rate of harmful or inadequate responses for ASP versus 0% for the chatbot (*p*= .497). However, M2 (Background Interpretation of Organism and Resistance Genes), had a significantly higher rate for the chatbot compared to ASP (4 v. 1%; *p*= .001), indicating that while the chatbot consistently identified BCID2 results correctly, it was more likely to provide incomplete or overly broad organism background information.

For Source Control Considerations, M3 (Emphasis on Source Control Importance) showed a low rate of harmful or inadequate responses that were not statistically different between the ASP and chatbot groups (0% v. 2%; *p*= .534). However, M4 (Organism-Specific Source Control Guidance) showed a larger difference, with harmful or inadequate responses occurring significantly more frequently for chatbot evaluations compared to r ASP (10% v. 2%; *p*= .022). This suggests that the chatbot was less reliable in providing organism-specific guidance for source control, potentially leading to incomplete management.

The largest discrepancy between the ASP and chatbot groups occurred in Antibiotic Therapy Recommendations. Evaluators classified 94% of ASP recommendations as optimal, compared to 63% for the chatbot. Harmful or inadequate recommendations occurred in 8% of chatbot evaluations for M5 (Optimal Treatment Selection) compared to 0% for ASP (*p*<.001), and in 10% v. 1% for M6 (Tailoring Treatment to Patient-Specific Factors) respectively (*p*= .022). The most common errors in the chatbot’s harmful responses involved: (1) inadequate antimicrobial coverage, particularly in complex cases with polymicrobial infections or resistant organisms; (2) incorrect dosing adjustments for renal impairment; (3) misdiagnosis of infection sources, leading to inappropriate antibiotic selection; (4) ambiguous treatment plans. Additionally, non-optimal chatbot recommendations were attributed to overly broad-spectrum antibiotic choices (35%, 15/43). While these regimens were not immediately harmful, they failed to align with best practices (Table 3). For Additional Diagnostic Workup (M7: Further Diagnostic Evaluation), chatbot recommendations were optimal in 75% of cases, compared to ASP’s 81%. However, inadequate responses were similar and not statistically significant (5% v. 4%; *p*= .68).

### Discussion

This study compared the performance of a GPT-powered chatbot with an ASP in interpreting BCID2 results and providing treatment recommendations. The chatbot demonstrated strong potential, particularly in its ability to accurately interpret BCID2 findings and generate structured responses. However, significant gaps remain in its performance, particularly in antibiotic therapy recommendations and source control considerations, where human expertise outperformed AI.

In an indirect comparison to a prior study, our chatbot’s rate of harmful and inadequate responses across all measures was 37% (16/43 cases) compared to 59% (26/44) in the previous study.^22^ For antibiotic therapy recommendations, harmful or inadequate responses occurred in 19% (8/43 cases) versus 36% (16/44) for ASP or ID consult providers in the previous study.^22^ Despite this relative improvement, the chatbot still underperformed in optimal antibiotic selection compared to the ASP. Most harmful responses involved inadequate antimicrobial coverage, particularly in complex cases with polymicrobial infections, co-infections, or contaminants. Additionally, the chatbot rarely recommended discontinuing antimicrobial therapy even when appropriate, likely due to difficulty in distinguishing true infections from contaminants. This may have been due to the chatbot receiving limited clinical information, such as the number of positive sets, which could have helped distinguish true infections from likely contaminants. Future iterations will require these data points to improve decision-making in such situations.

These shortcomings emphasize the need for refinement in COT reasoning and better integration of EKI, particularly in the interpretation of contaminants and polymicrobial infections. Beyond improvements in prompting and reference materials, LLMs may lack the intuitive pattern recognition and implicit knowledge that clinicians develop through experience, making it difficult to fully capture contextual subtleties and clinical judgment that guide real-world decision-making.

Within BCID2 interpretation, the chatbot performed well in identifying organisms and resistance genes but lacked contextualization often provided by human experts. In source control considerations, its recommendations were generalized rather than case-specific, making it less precise in complex infections that required targeted interventions. The additional diagnostic workup domain showed less pronounced differences, though the chatbot occasionally provided nonspecific or incomplete recommendations. Even without detailed context, BCID2 interpretation can still be valuable, especially when immediate ASP input is unavailable, such as during overnight coverage when quick decisions about empiric therapy are crucial and further diagnostics are left to other clinicians.

### Impact of prompting, external knowledge integration, and structured outputs

This study employed COT prompting, which improved response accuracy compared to previous studies that used standard prompting.^10,21,22,18,32^ This was done by customizing a GPT-powered chatbot by defining its purpose, refining its behavior with natural language instructions with COT prompting, and integrating external knowledge sources. COT’s step-by-step reasoning improved the user experience by eliminating the need for lengthy instructional prompts at the start of every case. Instead, the chatbot automatically introduced itself and requested key patient information before generating responses.

A key advancement of our chatbot, compared to previous studies, was its ability to integrate external knowledge by incorporating our local treatment guide developed from national antimicrobial treatment guidelines and primary literature. These additional uploads allowed the chatbot to reference evidence-based recommendations when formulating responses, aligning more closely with best practices for antimicrobial therapy. This approach helped mitigate a significant limitation with general LLMs that are not trained on biomedical data and subject matter expertise. The ability to reference evidence-based guidelines applied to a local context represents a critical strength of this study and demonstrates how LLMs can be customized for specific clinical decision support roles.

### Limitations

Several limitations must be considered. The single-center design and small sample size restricted the evaluation of less common organisms included in the BCID2 panel. Evaluators were not blinded to the source of recommendations (ASP v. chatbot), introducing potential bias. Clinical practice variability among ID specialists and evolving guidelines also complicate standardization of accuracy assessment. Only the ASP used the chatbot; therefore, we did not assess if non-ID clinicians would be able to effectively input the required patient characteristics and BCID2 results into the chatbot. A future study will evaluate the chatbot’s utility for non-ID clinicians, incorporating safeguards based on the study’s findings to ensure adequate input for optimal output and support clinical decision-making.

The chatbot’s responses were assessed in isolation, meaning no follow-up queries were permitted to refine its output. We did not assess reproducibility, and each case was entered once. Next, while COT prompting and EKI were implemented to reduce the risk of hallucinations, this study did not formally quantify hallucination frequency in chatbot-generated responses, though none were reported in the evaluations. Further studies are needed to assess how frequently hallucinations occur, their clinical impact, and whether additional refinements, such as real-time error detection mechanisms, could mitigate this risk.

## Conclusion

While the chatbot is not yet capable of replacing human oversight, its potential as a supportive tool in antimicrobial stewardship is clear. By mitigating resource constraints, standardizing initial assessments, and assisting in decision-making, AI could improve the efficiency and effectiveness of ASP interventions in the future. However, its clinical application must be approached cautiously, requiring rigorous validation, ongoing refinement, and an understanding of its limitations.

This study demonstrates that GPT-powered chatbots show promise as adjuncts in the management of bacteremia, particularly in BCID2 interpretation. However, challenges remain in antibiotic therapy recommendations and source control considerations, highlighting the need for more refined prompts, robust EKI, and the continued importance of human expertise. Future research should focus on refining AI models, integrating them into clinical workflows, and conducting larger, multicenter validation studies. This could include use of locally-trained biomedical LLMs with enhanced data security, specifically tailored for a healthcare system. With continued advancement, AI has the potential to complement human clinical judgment, improve patient outcomes, and optimize resource utilization in infectious disease management.

## Supporting information

10.1017/ash.2025.10059.sm001 Tassone et al. supplementary material Tassone et al. supplementary material

## Supplementary material

For supplementary material/s referred to in this article, please visit https://doi.org/10.1017/ash.2025.10059.

## Notes

Preliminary results from this study were presented as a poster at IDWeek 2024 (October 16–19, 2024, Los Angeles, California).
