# Supplementary material for: Evaluating chain-of-thought prompting in a GPT chatbot for BCID2 interpretation and stewardship: how does AI compare to human experts?
Source: Antimicrob Steward Healthc Epidemiol. 2025 Jul 11;5(1):e154. doi: 10.1017/ash.2025.10059 (PMC12247004; doi:10.1017/ash.2025.10059)
Supplement: Tassone et al. supplementary material [file S2732494X25100594sup001.docx]

**Evaluating Chain-of-Thought Prompting in a GPT Chatbot for BCID2 Interpretation and Stewardship: How Does AI Compare to Human Experts?**

**Supplementary Material**

The authors provide this appendix to offer readers additional information about their work.

Table of Contents

**Supplemental Tables**………………..………………………………………………………...3

**Table S1**. Chain-of-Thought Prompts……………...…………………..……………..3

**Table S2**. Examples of External Knowledge Integration Reference Documents...5

1. Empiric Treatment Options Based on BCID2 Results...........................5
2. BCID2 Result Interpretation and Additional Considerations..................6
3. Alternative Treatments due to Antibiotic Allergies..................................7

**Table S3**. Example of ChatGPT-4 Conversation with the Antimicrobial Stewardship Program…………………………………………………………………..8

**Table S4**. Study Definitions for Domains and Measures……………………...…..10

**Supplemental Figures**……………………………………………………………………….12

**Figure S1**. Example of a Mock ASP Blood Culture Notification Note……………12

**Supplemental Tables**

**Table S1**. Chain-of-Thought Prompts

| Initial Prompt | As the CVHCS BCID2 Treatment Advisor, immediately after my introduction, I will always prompt the user to enter the necessary information for analysis. I will prompt the user to enter the below information:  1. BCID2 Results: organism(s) and any resistance genes  2. Source of infection  3. Current antibiotics  4. Known antibiotic allergies  5. Presence of any hardware, chronic foleys, drains, or implants  6. Renal function  7. BMI  8. Does the patient have another infection not related to bacteremia, if so then what is the treatment? |
| --- | --- |
|  | Upon receiving the BCID2 results and additional patient details, my responses will follow a structured format in bullets that can be a few sentences in length if needed to explain:  1. BCID2 Result Interpretation:  a. This will include the interpretation of organism(s) and resistance genes when identified.  b. I will always use the information from file 'BCID2 Result Interpretation and Additional Considerations,' to provide the interpretation of the results and include the bullet points for the specific organism and resistance gene identified when applicable. |
| BCID2 Result Interpretation | 1. BCID2 Result Interpretation:  a. This will include the interpretation of organism(s) and resistance genes when identified.  b. I will always use the information from file 'BCID2 Result Interpretation and Additional Considerations,' to provide the interpretation of the results and include the bullet points for the specific organism and resistance gene identified when applicable. |
| Source Control | 3. Source Control:  a. Emphasize the importance of source control to cure infection. Suggest organism-specific sources of infection that should be investigated when applicable. This information may also be found in the file 'BCID2 Result Interpretation and Additional Considerations.'  b. Discuss the importance of controlling the infection source before narrowing antibiotic treatment, especially in cases of polymicrobial sources such as foot infections, intra-abdominal infections and abscess, perforated bowel, necrotizing fasciitis, sacral decubitus ulcers, cholangitis, liver abscess, gastrointestinal infections (not UTIs), or other intra-abdominal sources where anaerobic coverage maybe empirically important.  c. The above polymicrobial infections may require empiric gram-positive and gram-negative coverage until all cultures finalize. Therefore, it may not be safe to narrow therapy at this time. d. Clarify that repeat blood cultures are not required for Gram-negative organisms. |
| Antibiotic Treatment  Recommendations | 2. Treatment:  a. Provide an optimal treatment regimen based on the identified organism and resistance gene, including medication(s), dose, and escalation or de-escalation of current antibiotic treatment when applicable.  Suggest alternative options for patient-specific factors (antibiotic allergies, obesity, renal impairment) when applicable.  I will only use the information in the file 'Table 1 Empiric Treatment Options Based on BCID2 Results,' and from the file 'Table 2 Alternative treatments due to antibiotic allergy,' for specific organism, resistance, and allergy treatment recommendations.  b. Include guidance on alternatives for patients with drug allergies, emphasizing that patients with a penicillin allergy may still use cefazolin, given the difference in the R1 side chain, typically resulting in no cross-reactivity, and to avoid nafcillin due to its similar structure to penicillin, which may result in cross-reactivity. There is no cross-reactivity expected with meropenem and other beta-lactams like penicillin, cefazolin, ceftriaxone, or piperacillin-tazobactam. Clarify that ceftriaxone does not require renal dosing and is always dosed at 2 grams IV every 24 hours.  c. If the BCID2 result is Streptococcus genus with all other Streptococcus species NEGATIVE (i.e.: S. agalactiae, S. pneumoniae, and S. pyogenes), then I will ask the following question before giving my full recommendation: 'How many blood culture bottles were positive?'  d. If the BCID2 result is Staphylococcus genus (other than S. aureus, S. lugdunensis), then I will ask the following question before giving my full recommendation: 'How many blood culture bottles were positive?'  e. I can refer to the file called 'CoNS algorithm' if needed for additional guidance about CoNS.  f. I will never recommend less than ceftriaxone 2 grams IV every 24 hours.  g. For Stenotrophomonas maltophilia, always recommend sulfamethoxazole/trimethoprim (Bactrim) and minocycline together. |
| Addition Workup | 4. Additional Considerations:  a. Suggest additional diagnostic workup when applicable.  b. I will use the information for the specific organism and resistance gene in the file 'BCID2 Result Interpretation and Additional Considerations.'  c. I will present my answers in bullet points. |
| Disclaimer | 5. Disclaimer:  a. I will always provide this disclaimer after giving my recommendations: 'This note is based on chart review and DOES NOT REPRESENT a formal consultation. Please page the antimicrobial stewardship team at XXXX with any questions. If a formal infectious disease consult is needed, please page XXXX.'  b. This statement will be underneath the bullet points.  c. Then I will add this text at the end of the conversation: 'The BCID2 Panel is indicated as an aid in the diagnosis of specific agents of bloodstream infections and results should be used in conjunction with clinical history, signs and symptoms, and results of other diagnostic tests including Gram stain, culture, and antimicrobial susceptibility testing. A negative result does not exclude the possibility of a bloodstream infection. Positive results do not rule out coinfection with organisms not included on the BCID2 panel.  ' I may refer to the file 'Frequently Asked Questions Background and Limitations' if the user asks additional questions about what the BCID2 is, frequently asked questions, and limitations. |

**Table S2**. Examples of External Knowledge Integration reference documents

**A.** Empiric Treatment Options Based on BCID2 Results

| **BCID2 results** | **Preferred therapy** |
| --- | --- |
| **Gram-positive organisms** | |
| ***Enterococcus faecalis***  van A/B negative | Ampicillin 2 g IV every 4 hours (requires renal dosing) |
| ***Enterococcus faecalis***  van A/B positive: VRE (Uncommon) | Ampicillin 2 g IV every 4 hours (requires renal dosing) |
| ***Enterococcus fecium***  van A/B negative | Vancomycin (PharmD dosing with trough goal 15-20: initiate with a loading dose) |
| ***Enterococcus fecium*** van A/B positive=VRE (Common) | Daptomycin 8-12 mg/kg every 24 hours (requires renal dosing, if BMI > 30 then use Adjusted Body weight instead of actual body weight for dosing, this drug requires ASP approval) |
| ***Staphylococcus* genus** (other than *S. aureus, S. lugdunensis*) | See Coagulase Negative *Staphylococci* flowchart below |
| ***Staphylococcus aureus***  mecA/C and MREJ negative = MSSA | Cefazolin 2 g IV every 8 hours (requires renal dosing) |
| ***Staphylococcus aureus***  mecA/C and MREJ positive = MRSA | Vancomycin (PharmD dosing with trough goal 15-20 initiate with a loading dose) |
| ***Staphylococcus lugdunensis***  mecA/C negative = oxacillin susceptible | Cefazolin 2 g IV every 8 hours (requires renal dosing) |
| ***Staphylococcus lugdunensis***  mecA/C positive = oxacillin resistant | Vancomycin (PharmD dosing with trough goal 15-20, initiate with a loading dose) |
| ***Streptococcus* genus with all other *Streptococcus* species NEGATIVE** (ie*: S. agalactiae, S. pneumoniae, and S. pyogenes*)  **Blood culture bottles positive: only 1** | Consider withholding therapy if only 1 out of 2 bottles positive with viridians group streptococci, as this may be considered contaminant in correct clinical setting |
| ***Streptococcus* genus with all other *Streptococcus* species NEGATIVE** (ie*: S. agalactiae, S. pneumoniae, and S. pyogenes*)  **Blood culture bottles positive: 2 or more** | Ceftriaxone 2 g IV every 24 hours |
| ***Streptococcus pyogenes* (Group A strep) and *Streptococcus agalactiae* (Group B strep)** | Ceftriaxone 2 g IV every 24 hours |

**B.** Result Interpretation and Additional Considerations

| Staphylococcus aureus | Staphylococcus aureus should always be considered a serious pathogen. It may cause a broad range of clinical manifestations including skin and soft tissue infections, endocarditis, cardiac device infections, catheter-related bloodstream infections, pneumonia, osteomyelitis, and musculoskeletal infections to name a few.  -Conduct a thorough physical examination to assess for sources  (cardiac exam for new murmurs suggesting endocarditis, skin  exam assessing for cellulitis/abscess and peripheral stigmata of  endocarditis, joint exam for signs of inflammation, palpation of  spine to assess for vertebral or paravertebral involvement,  presence of intravascular catheters/devices/prosthetic joints, etc...)  -Repeat blood cultures every 24-48 hours until clear  -Perform a TTE and if clinically indicated a TEE  -Pursue source control if indicated (e.g, drainage of abscess,  removal of infected central line)  -Consider infectious disease consultation (pager 9886)  -If mecA/C and MREJ positive = MRSA: This gene codes for  resistance to cefazolin and oxacillin.  -Begin treatment with VANCOMYCYIN.  -If mecA/C and MREJ negative = MSSA:  -Begin treatment with NAFCILLIN (preferred) or CEFAZOLIN. |
| --- | --- |
| Coagulase negative Staphylococcal | Coagulase negative Staphylococci (CoNS) are considered normal flora of human skin. CoNS bacteremia is often a contaminant, but in specific situations CoNS may cause a true infection.  -If the patient has NONE of the following risk factors, would  consider observation OFF antibiotics:  -Fever, leukocytosis, shock, tachycardia (clinical signs  of infection)  -Concern for infection at a local catheter site  -Presence of an intravascular device (e.g., AICD, pacemaker,  prosthetic joint, prosthetic valves)  -Multiple sets of positive blood cultures  -History of solid organ or bone marrow transplant  -Neutropenia  -Immunocompromised for other reasons  -Active IV drug use  -If the patient has any of the above risk factors, it is reasonable  to obtain 2 additional sets of blood cultures, THEN begin empiric  antibiotics with VANCOMYCIN.  -Optimal duration of antimicrobial therapy depends on source  control and degree of invasiveness of infection. For uncomplicated  infection, 7-14 days of therapy is likely adequate if no evidence of  endocarditis; no implanted prostheses; follow-up blood cultures are  negative; and no evidence of metastatic sites of infection. |

**C.** Alternative Treatments due to Antibiotic Allergies

If a patient is currently tolerating the medication that they have a reported allergy to, or if they recently had the medication in the past with no reactions, then they can continue the medication that has a reported allergy. Example: a person with a past allergy to penicillin (PCN) or ampicillin is now receiving the medication and tolerating it without any adverse drug reaction may continue to take the medication. There is no need to switch to an alternative therapy.

| **Allergy to preferred therapy** | **Alternative therapy** | **Comment** |
| --- | --- | --- |
| Penicillin (PCN), ampicillin, nafcillin, oxacillin | Vancomycin | Patient’s with *Acinetobacter baumannii* complex and Ampicillin allergy should not be switched to vancomycin. It is recommended to consult ID for alternative therapy. |
| Vancomycin | Daptomycin |  |
| Cefazolin | Vancomycin for Staphylococci infections, but use Piperacillin/Tazobactam for E. coli infections. | cefazolin does not have a cross allergy to ceftriaxone |
| Ceftriaxone | Piperacillin/Tazobactam | cefazolin does not have a cross allergy to ceftriaxone |
| Cefepime | Piperacillin/Tazobactam if not a potential AmpC producing organism, if so then recommend meropenem.  Or Meropenem | Do not recommend ceftriaxone if allergy to cefepime. |
| Meropenem | ID Consult required | Likely drug resistant organism and ID consult required for review. |
| Daptomycin | If Daptomycin is the preferred therapy like with VRE, then use Linezolid.  If the pathogen is susceptible to Vancomycin, then vancomycin can be used as the alternative |  |
| Sulfamethoxazole/Trimethoprim | ID Consult required | For *Stenotrophomonas maltophilia*  Ceftazidime avibactam with Aztreonam |

**Table S3**. Example of ChatGPT-4 conversation with the Antimicrobial Stewardship Program

| **Chatbot** | Please provide the following information for analysis:   1. BCID2 Results: Organism(s) identified and any resistance genes detected 2. Source of Infection 3. Current Antibiotics 4. Known Antibiotic Allergies 5. Presence of Hardware, Chronic Foley, Drains, or Implants 6. Renal Function (e.g., SCr, CrCl, or AKI status) 7. BMI 8. Does the patient have another infection not related to bacteremia? If so, what is the current treatment?   Once you provide these details, I can give specific recommendations. |
| --- | --- |
| **User** | E. coli with no resistance, possible urinary source, chronic foley, PCN allergy, on piperacillin/tazobactam x 2 days, moderate renal impairment |
|  | BCID2 Result Interpretation:   - Escherichia coli detected with no resistance genes identified. - E. coli is a common cause of bloodstream infections, often originating from urinary tract infections (UTIs), intra-abdominal infections, or biliary infections.   Source Control:   - The suspected source is the urinary tract, and the presence of a chronic Foley catheter suggests a catheter-associated UTI (CA-UTI) as a potential source. - Chronic Foley catheter should be replaced if not already done, as this is likely the source of infection.   Treatment Recommendations:   - De-escalation is recommended, as no resistance genes were detected, and piperacillin/tazobactam is unnecessarily broad. - Recommended Treatment:   - Ceftriaxone 2 g IV every 24 hours (preferred; safe despite PCN allergy and does not require renal dose adjustment).   - Penicillin (PCN) Allergy Consideration:     - Ceftriaxone is safe to use despite a penicillin allergy, as it does not share the same R1 side chain as penicillins, meaning there is no expected cross-reactivity.   - If unable to use ceftriaxone due to intolerance or other concerns:     - Piperacillin/tazobactam (patient tolerating for the last 48 hours).     - Ertapenem 1 g IV every 24 hours (only if ESBL emerges in final susceptibilities but not needed here).   Additional Considerations:   - Consider obtaining a urine culture to confirm the source and guide further therapy. - Repeat blood cultures are not needed for Gram-negative bloodstream infections unless there is clinical concern for persistent bacteremia. - Duration of therapy: Typically 7–14 days, depending on clinical improvement and resolution of bacteremia.   This note is based on chart review and DOES NOT REPRESENT a formal consultation. Please page the antimicrobial stewardship team at XXXX with any questions. If a formal infectious disease consult is needed, please page XXXX.  The BCID2 Panel is indicated as an aid in the diagnosis of specific agents of bloodstream infections and results should be used in conjunction with clinical history, signs and symptoms, and results of other diagnostic tests including Gram stain, culture, and antimicrobial susceptibility testing. A negative result does not exclude the possibility of a bloodstream infection. Positive results do not rule out coinfection with organisms not included on the BCID2 panel. |

**Table S4**. Study Definitions for Domains and Measures

| **Domain** | **Measures (M)** | **Definitions** |
| --- | --- | --- |
| **BCID2 Results Interpretation** | **M1:** Appropriately interprets organism and resistant gene targets when identified.  **M2:** Appropriately provides general background information about the organism and resistant gene targets when identified. | This domain evaluated the chatbot's and ASP’s ability to accurately interpret BCID2 panel findings, including organism identification and detection of resistance gene targets. Interpretations were scored as appropriate if they correctly identified the organism and resistance genes while providing accurate and relevant background information. This information included the types of infections typically caused by the organism, including common sources, and the implications of identified resistance genes. Interpretations were considered not specific enough or overly broad if they lacked the detail necessary for optimal clinical utility or educational value but were not clearly harmful. Failure to identify the correct organism or resistance gene were categorized as Inadequate or incorrect interpretations due to the high potential for patient harm. |
| **Source Control Considerations** | **M3:** Appropriately emphasizes the importance of source control to cure infection.  **M4:** Suggests organism-specific sources of infection that should be investigated when applicable. | Source control considerations emphasized a critical component of infection management, particularly in identifying and addressing the infection’s origin and associated foci. Recommendations were considered appropriate when they thoroughly addressed the importance of source control and included organism-specific guidance, such as evaluating and removing intravascular catheters in *Staphylococcus aureus* bacteremia. Recommendations that offered vague or generic advice that lacked actionable steps tailored to the organism and infection were categorized as not specific enough, overly broad, or general. Inadequate or incorrect recommendations either omitted meaningful source control measures or included suggestions inappropriate for the identified organism, potentially leading to patient harm. |
| **Antibiotic Treatment** | **M5**: Appropriately suggests optimal treatment regimen based on the identified organism including medication, dose, and escalation versus de-escalation (when applicable).  **M6:** Appropriately suggests alternative options for patient specific factors (e.g., antibiotic allergies, renal impairment, obesity) when applicable. | This domain evaluated the appropriateness of antimicrobial treatment recommendations based on the identified organism including medication, dose, and spectrum of therapy. Additionally, it assessed the appropriateness of alternative treatment options based on patient-specific factors such as the presence of an antibiotic allergy, renal impairment, or obesity. Optimal recommendations aligned with the identified organism and resistance genes, providing correct dosing and route of administration. These suggestions balance effective coverage while minimizing unnecessary antimicrobial exposure. For example, a beta-lactam antibiotic dosed appropriately for renal function in methicillin-susceptible *Staphylococcus aureus* bacteremia would be considered optimal. Non-optimal recommendations provided adequate coverage but were not the preferred regimen, such as overly broad-spectrum therapies or regimens that failed to adhere to best practices (e.g., vancomycin for methicillin-susceptible *Staphylococcus aureus* bacteremia in the absence of a beta-lactam allergy). While these recommendations were not actively harmful, they were suboptimal. Inadequate or harmful recommendations failed to provide sufficient coverage, incorrect dosing, unclear or confusing recommendations (e.g., did not clearly state to discontinue a current antibiotic prior to escalating therapy), or posed risks such as missing allergies or causing treatment delays. Examples include suggesting antibiotics without activity against the identified organism or failing to adjust dosing for renal insufficiency. |
| **Additional Diagnostic Workup** | **M7:** Appropriately suggests additional diagnostic workup when applicable. | This domain examined whether appropriate additional diagnostic evaluations were suggested to further clarify the infection’s source or guide treatment. Recommendations were deemed appropriate if they aligned with best practices and addressed the need for further imaging, cultures, or other laboratory tests, such as recommending an echocardiogram for suspected endocarditis in *Staphylococcus aureus* bacteremia. Recommendations were classified as not specific enough, overly broad, or general, if they were valid but lacked actionable detail (e.g., suggesting "consider imaging" without specifying a modality or rationale). Lastly, a recommendation was considered inadequate or incorrect if it omitted necessary diagnostic steps, including inappropriate or unnecessary suggestions. An example of this is failing to recommend imaging for suspected osteomyelitis or suggesting unwarranted invasive procedures. |

**Supplemental Figures**

**Figure S1**. Example of a Mock ASP Blood Culture Notification Note

ID BLOOD CULTURE NOTE (E-CONSULT)

Antimicrobial Stewardship Team

************************************************************************************************************************

This note is based on chart review and DOES NOT REPRESENT a formal consultation. Please page the antimicrobial stewardship team at XXXX with any questions. If a formal infectious disease consult is needed, please page XXXX.

************************************************************************************************************************

CC: Bacteremia

HPI: 74 year old male with dementia, hypertension, type 2 diabetes, CKD2, BPH, prior MI who presented to the emergency department with altered mental status, increase in urine output, and chills. Blood cultures drawn in the ED resulted positive for GNRs. Patient has an indwelling catheter that was changed in the ED. Patient had leukocytosis on admission, with 510 WBC in his UA. Likely source of bacteremia is UTI. Allergy to PCN. CrCl of 42. BMI 31.

================================================================================

CURRENT ANTIMICROBIALS:

- Piperacillin/tazobactam 3.375g IV q8h
- Vancomycin 2000mg IV once while in the ED

================================================================================

MICROBIOLOGIC DATA:

- Blood x 2: 1/4 bottles GNR
- Urine: pending
- MRSA nares: negative

BCID2 Result:

The BioFire Blood Culture Identification 2 (BCID2) Panel is a multiplex, in vitro diagnostic test designed for qualitative detection and identification of multiple bacteria and yeast nucleic acids and select antimicrobial genes from blood culture samples identified as positive.

BCID2 Identified: Gram Negative organisms:

- Enterobacterales Order
- Escherichia coli
  - NO Resistance Genes Detected (e.g. CTX-M)

================================================================================

ASSESSMENT AND RECOMMENDATIONS:

- If source presumed to be a MONOMICROBIAL infection, attempt to optimize antibiotic therapy to target organism(s) identified by BCID2.
- If source presumed to be POLYMICROBIAL (e.g., intra-abdominal abscess, perforated bowel, diabetic foot infection, necrotizing fasciitis, infected sacral decubitus ulcer, cholangitis, liver abscess, gastrointestinal infection, cholecystitis, or other intra-abdominal source), WOULD NOT narrow antibiotics until the source has been fully controlled.
- Possible/potential SOURCE of infection based on documentation: UTI
- Efforts to control the source of infection (i.e., removal of infected devices/lines/hardware, drainage of abscess) should be considered when applicable to optimize chance of cure.
- Bacteremia: Escherichia coli
  - E. coli is part of the normal flora of the GI tract and is most commonly associated with gastrointestinal and genitourinary infections, central line infections, and wound infections. E. coli can also cause more invasive infections such as meningitis and septic arthritis.
  - For patients who clinically improve after the initiation of appropriate antibiotic therapy, repeat blood cultures to document clearance of bacteremia may be unnecessary.
  - Duration of therapy should be determined by the clinical response of the patient in addition to the primary source and extent of infection. In most cases, the duration of antibiotic therapy is 7 to 14 days.
  - Patient had his foley changed 2 days ago and likely has source control.
  - CTX-M negative organism: begin treatment with CEFTRIAXONE. Patient lives at home, no recent antibiotics, and chart review indicates no history of MDROs in the past year.
  - Medicine team to follow Urine and blood culture results.
  - Reasonable to de-escalate therapy to CEFTRIAXONE 2g IV q24h
    - Discontinue: Piperacillin/tazobactam
    - Do not restart vancomycin
    - Patient with PCN allergy but tolerating current beta-lactam with piperacillin/tazobactam.
    - There is **less than 1%** rate of cross-reactivity with third-gen cephalosporins like ceftriaxone and PCN allergies.

************************************************************************************************************************

This note is based on chart review and DOES NOT REPRESENT a formal consultation. Please page the antimicrobial stewardship team at XXXX with any questions. If a formal infectious disease consult is needed, please page XXXX.

************************************************************************************************************************

The BCID2 Panel is indicated as an aid in the diagnosis of specific agents of bloodstream infections and results should be used in conjunction with clinical history, signs and symptoms, and results of other diagnostic tests including Gram stain, culture, and antimicrobial susceptibility testing. A negative result does not exclude the possibility of a bloodstream infection. Positive results do not rule out coinfection with organisms not included on the BCID2 panel.
